# Supplementary material for: A facile dual-template-directed successive assembly approach to hollow multi-shell mesoporous metal–organic framework particles
Source: Nat Commun. 2023 Dec 5;14:8062. doi: 10.1038/s41467-023-43259-2 (PMC10698178; doi:10.1038/s41467-023-43259-2)
Supplement: Supplementary file 1 — Supplementary Information [file 41467_2023_43259_MOESM1_ESM.pdf]

Supplementary Information

A facile dual-template-directed successive assembly approach to hollow multi-shell mesoporous metal–organic framework particles

Xu et al

Table of Contents

Supplementary Methods

Supplementary Figures

Supplementary Tables

Supplementary Methods

Synthesis of Hf-UiO-66-NH_2 dual-mesopore core-shell particles. Typically, 50 mg of ODMB and 50 mg of F127 were dissolved in 9.0 mL of deionized water, and then 0.8 mL of formic acid was added. The mixture was stirred for 15 min to form a homogeneous solution. Subsequently, 50 mg of $HfCl_4$ and 50 mg of BDC-NH_2 were added into above mixture. Then, the mixture was stirred at 45 °C for 20 h. The products were collected through centrifugation and then washed with DMF and ethanol. Finally, the products were redispersed in 2.0 mL of H_2O for further use (suspension H).

Synthesis of hollow single-shell mesoporous Hf-UiO-66-NH_2 particles. In a typical procedure, 1.0 mL of suspension H was well dispersed in a solution composed of 8.0 mL of H_2O and 1.0 mL of acetic acid by magnetic stirring. After 2 h of stirring at 25 °C, the products were collected by centrifugation and washed several times in DMF and ethanol. After dried at 80 °C for 12 h, the hollow single-shell mesoporous Hf-UiO-66-NH_2 particles were obtained.

Synthesis of hollow double-shell mesoporous Hf-UiO-66-NH_2 particles. Briefly, 70 mg of ODMB, 50 mg of F127, 0.05 g of glycerol, and 1.0 mL of acetic acid were dissolved in 8.0 mL of deionized water. Afterwards, 80 mg of $HfCl_4$, 1.0 mL of suspension H as seeds, 0.05 mL of n-hexadecane, and 50 mg of BDC-NH_2 were added to the reaction system. The obtained mixture was continuously stirred at 50 °C for 15 h. The product MOFs with double inhomogeneous layers was separated by centrifugation, washed with DMF and ethanol several times. The hollow double-shell mesoporous Hf-UiO-66-NH_2 particles were synthesized by chemical etching of corresponding MOF precursor particles with acetic acid.

Synthesis of MOF-801 dual-mesopore core-shell particles. Typically, 75 mg of ODMB and 75 mg of F127 were dissolved in 6.0 mL of deionized water, and then 1.5 mL of formic acid was added. The mixture was stirred for 15 min to form a homogeneous solution. Subsequently, 120 mg of $ZrOCl_2 \cdot 8H_2O$, 0.15 mL of n-hexadecane, and 45 mg of fumaric acid were added into above mixture. Then, the mixture was stirred at 45 °C for 5 h. The products were collected through centrifugation and then washed with DMF and ethanol. Finally, the products were redispersed in 2.0 mL of H_2O for further use (suspension M).

Synthesis of hollow single-shell mesoporous MOF-801 particles. In a typical procedure, 1.0 mL of suspension M was well dispersed in a solution composed of 8.0 mL of H_2O and 1.0 mL of formic acid by magnetic stirring. After 1 h of stirring at 25 °C, the products were collected by centrifugation and washed several times in DMF and ethanol. After dried at 80 °C for 12 h, the hollow single-shell mesoporous MOF-801 particles were obtained.

Synthesis of hollow double-shell mesoporous MOF-801 particles. Briefly, 75 mg of ODMB, 75 mg of F127, 0.15 g of glycerol, and 1.5 mL of formic acid were dissolved in 5.0 mL of deionized water. Afterwards, 120 mg of $ZrOCl_2 \cdot 8H_2O$, 1.0 mL of suspension M as seeds, 0.15 mL of n-hexadecane, and 45 mg of fumaric acid were added to the reaction system. The obtained mixture was continuously stirred at 45 °C for 5 h. The product MOFs with double inhomogeneous layers was separated by centrifugation, washed with DMF and ethanol several times. The hollow double-shell mesoporous MOF-801 particles were synthesized by chemical etching of corresponding MOF precursor particles with formic acid.

Synthesis of Au@3S-mesoUiO-66-NH_2 HoMSs. The Au@3S-mesoUiO-66-NH_2 HoMSs were obtained by mixing 0.10 g of 3S-mesoUiO-66-NH_2 HoMSs with 10 mg of $HAuCl_4$ in 40 mL of MeOH.

The resulting mixture was stirred at room temperature for 6 hours under a nitrogen atmosphere. Then, 0.20 g of $NaBH_4$ was added and stirred for an additional hour at room temperature. The resulting mixture was centrifuged to obtain Au@3S-mesoUiO-66-NH_2 HoMSs.

Hydrogenation reaction of nitrobenzene. A sodium borohydride solution (1.0 mL, 15 mM) was added to a nitrobenzene solution (10 mL, 1.46 mM) contained in a glass vessel. Thereafter, 25 mg of Au@3S-mesoUiO-66-NH_2 HoMSs was added to the solution. The mixture was stirred at room temperature for 1 h. After the reaction completion, UV/vis spectra of the sample were taken in the range of 225-400 nm for reactants and products.

Supplementary Figures

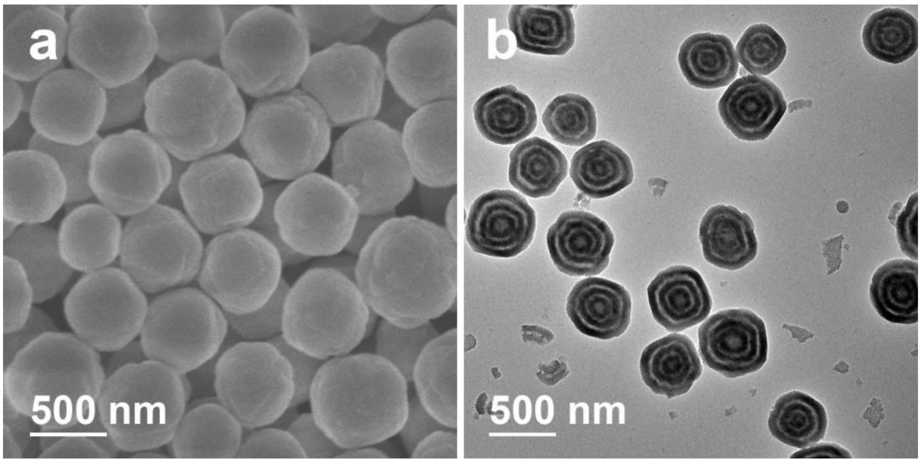

Supplementary Fig. 1 Low-magnification (a) SEM and (b) TEM images of 3S-mesoUiO-66-NH_2 HoMSs.

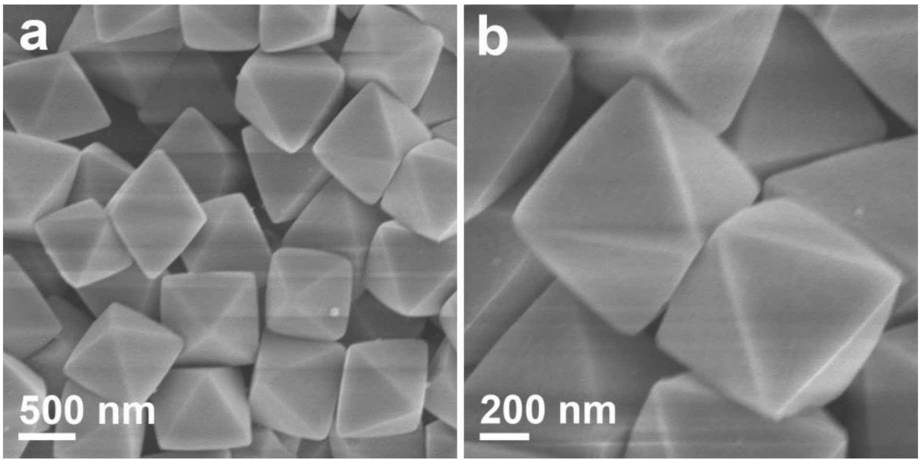

Supplementary Fig. 2 (a) SEM and (b) magnified SEM images of microporous UiO-66-NH_2 crystals.

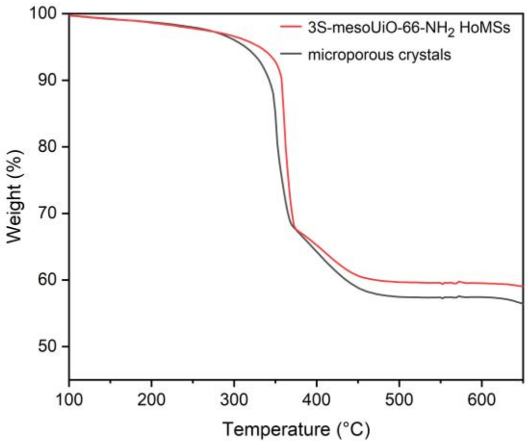

Supplementary Fig.3. TG curves of 3S-mesoUiO-66-NH_2 HoMSs and microporous UiO-66-NH_2 crystals.

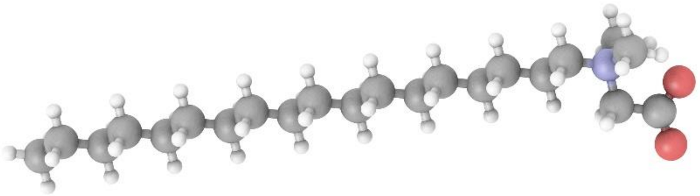

Supplementary Fig. 4 Chemical structure of the ODMB ($C_{18}H_{37}N^+(CH_3)_2CH_2COO^-$).

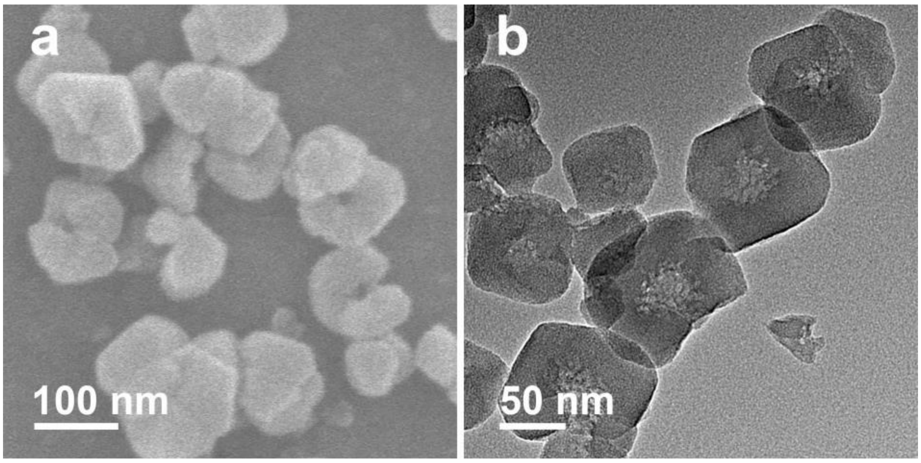

Supplementary Fig. 5 (a) SEM and (b) TEM images of UiO-66-NH_2 particles prepared with ODMB templates.

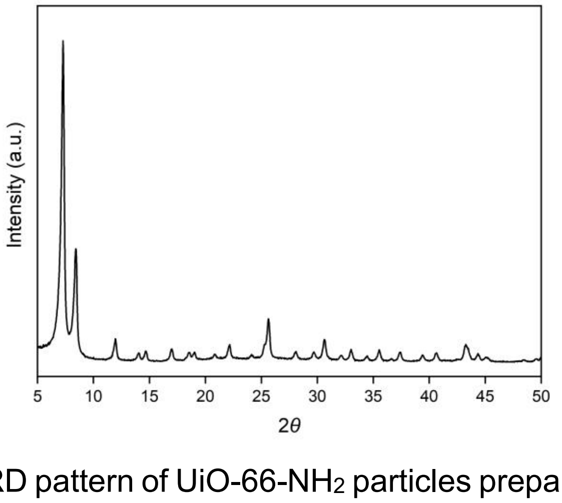

Supplementary Fig. 6 XRD pattern of UiO-66-NH_2 particles prepared with ODMB templates.

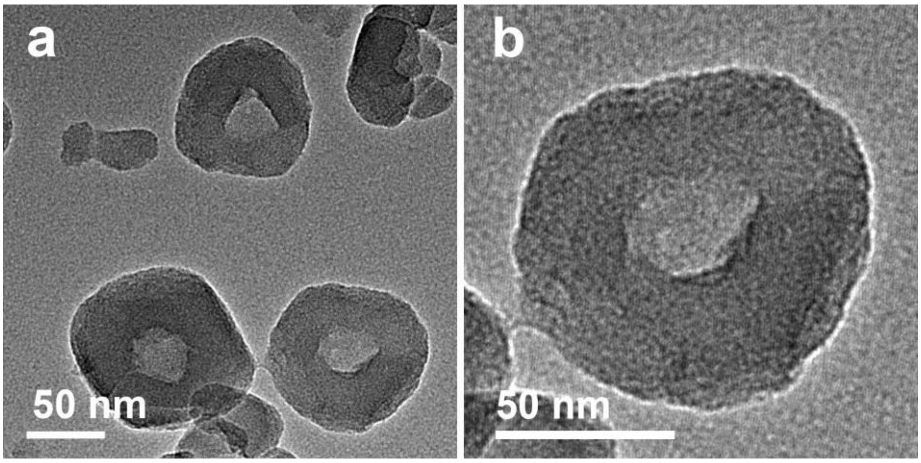

Supplementary Fig. 7 (a) TEM and (b) magnified TEM images of MOF hollow nanostructures obtained by etching of UiO-66-NH_2 particles prepared with ODMB templates by 5.25 M acetic acid solution.

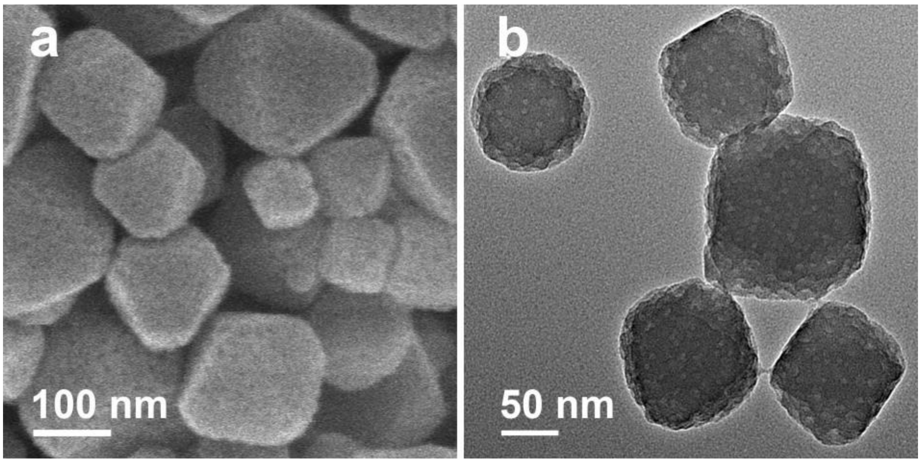

Supplementary Fig. 8 (a) SEM and (b) TEM images of UiO-66-NH_2 particles prepared with F127 templates.

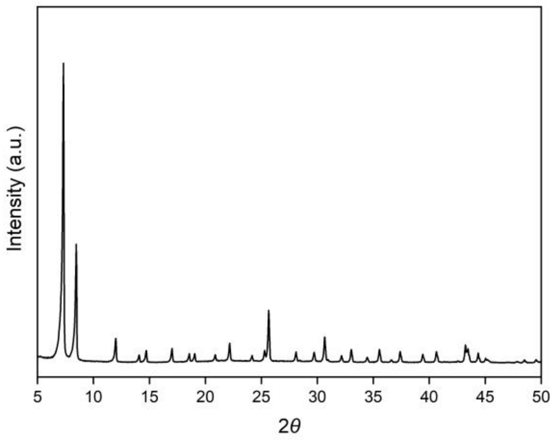

Supplementary Fig. 9 XRD pattern of UiO-66-NH_2 particles prepared with F127 templates.

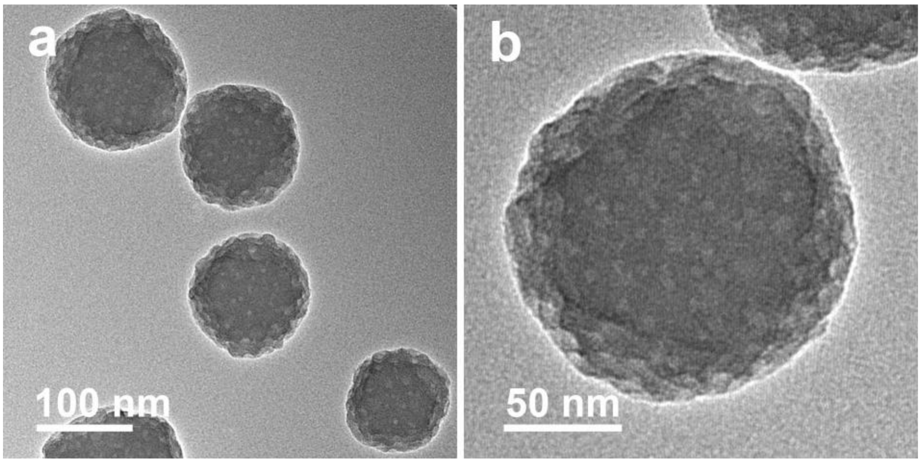

Supplementary Fig. 10 (a) TEM and (b) magnified TEM images of UiO-66-NH_2 particles prepared with F127 templates after treatment by 5.25 M acetic acid solution.

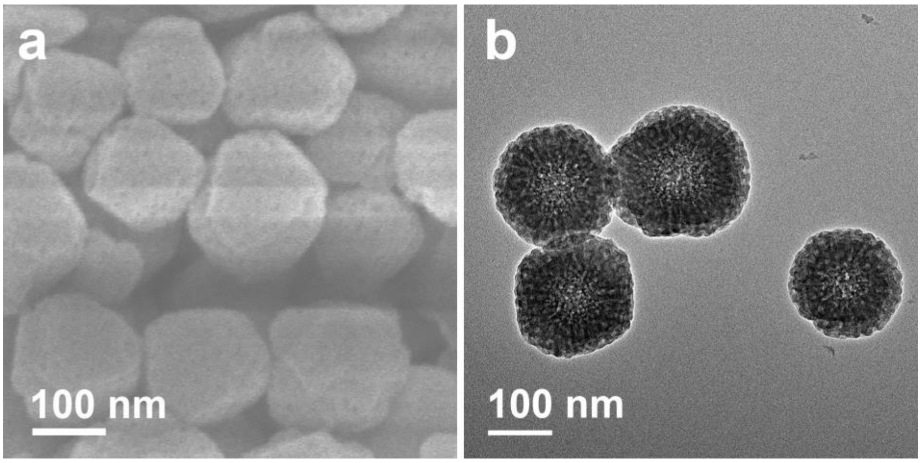

Supplementary Fig. 11 (a) SEM and (b) TEM images of UiO-66-NH_2 precursor particles prepared with both ODMB and F127 templates.

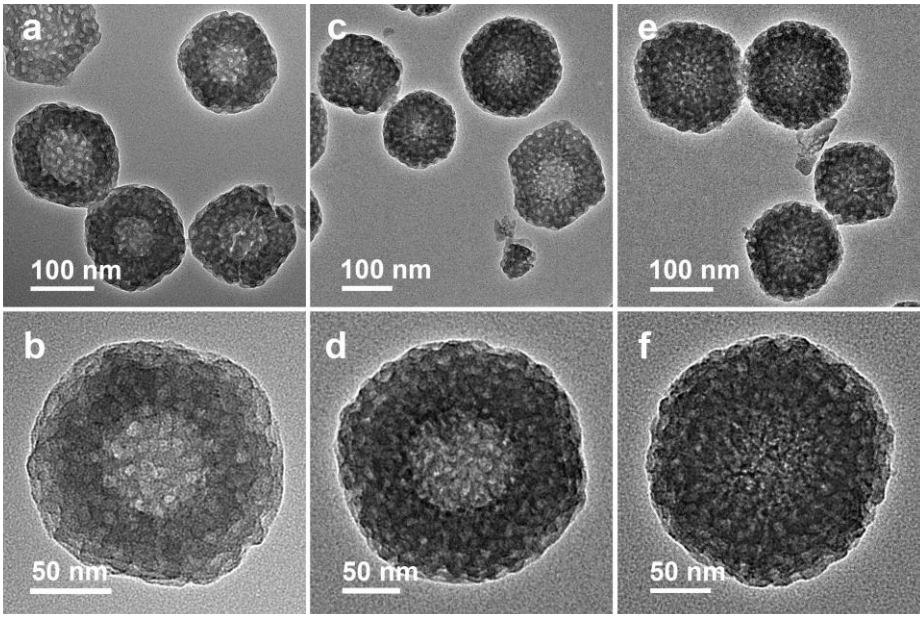

Supplementary Fig. 12 TEM and magnified TEM images of MOF nanostructures obtained by etching of UiO-66-NH_2 precursor particles prepared with ODMB and F127 templates in (a,b) formic acid, (c,d) propanoic acid, and (e,f) hydrochloric acid solution.

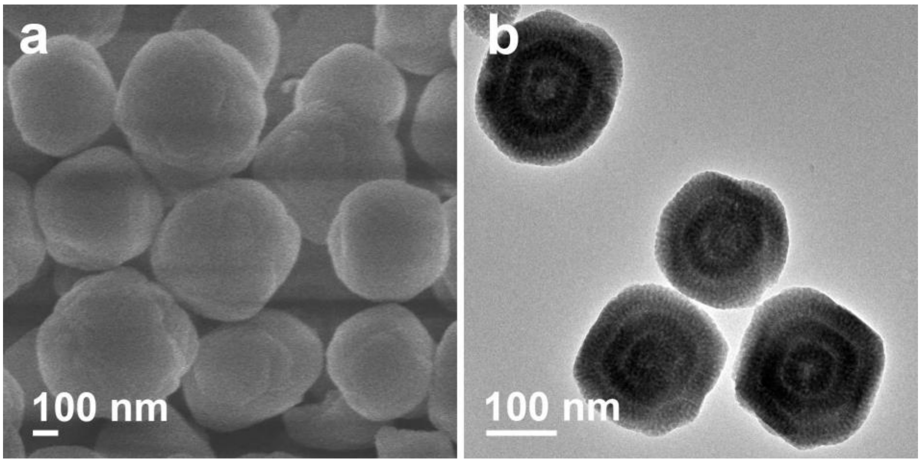

Supplementary Fig. 13 (a) SEM and (b) TEM images of UiO-66-NH_2 precursor particles with triple inhomogeneous layers consisting of two different mesostructured MOFs.

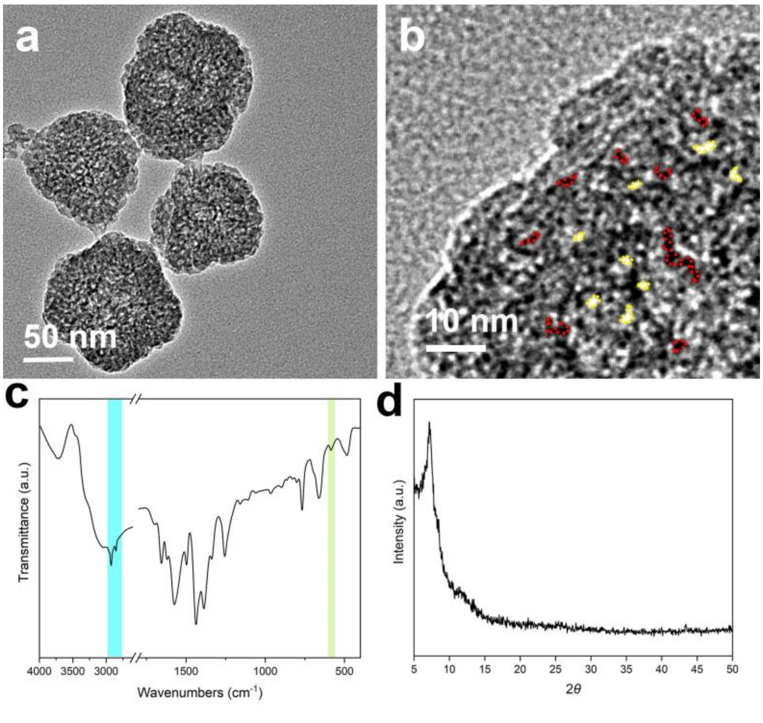

Supplementary Fig. 14 (a,b) TEM images, (c) FTIR spectrum, and (d) XRD pattern of the UiO-66-NH_2 particles prepared with ODMB and F127 templates at 4 h.

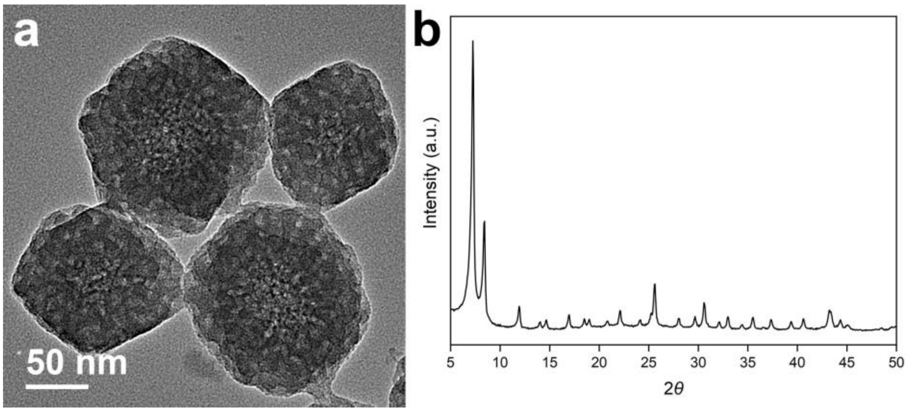

Supplementary Fig. 15 (a) TEM image and (b) XRD pattern of the UiO-66-NH_2 particles prepared with ODMB and F127 templates at 7 h.

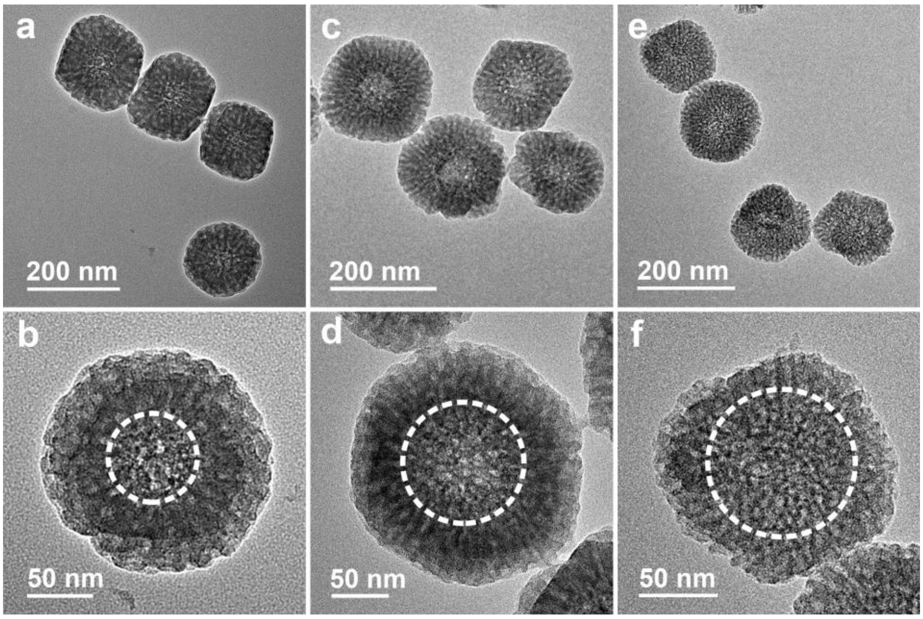

Supplementary Fig. 16 TEM and magnified TEM images of dual-mesopore core-shell UiO-66-NH_2 particles prepared with 0.05 g of F127 but different amount of ODMB: (a,b) 0.05 g, (c,d) 0.1 g, and (e,f) 0.2 g.

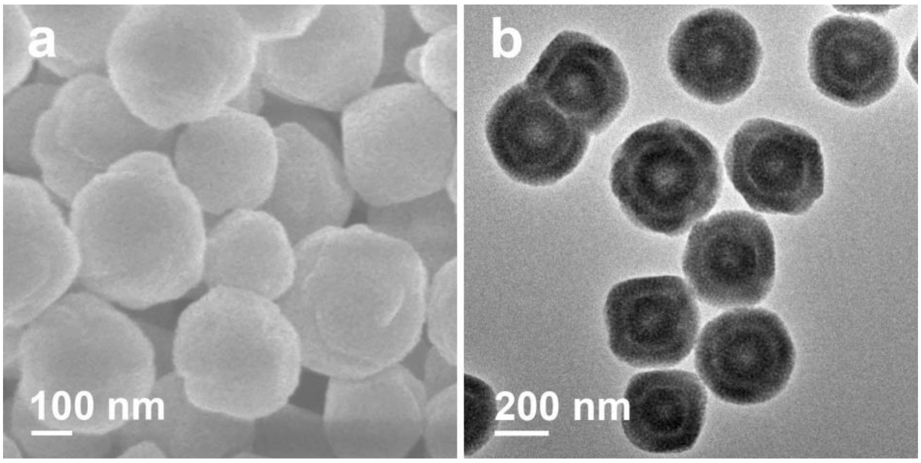

Supplementary Fig. 17 (a) SEM and (b) TEM images of UiO-66-NH_2 precursor particles with double layers consisting of two different mesostructured MOFs.

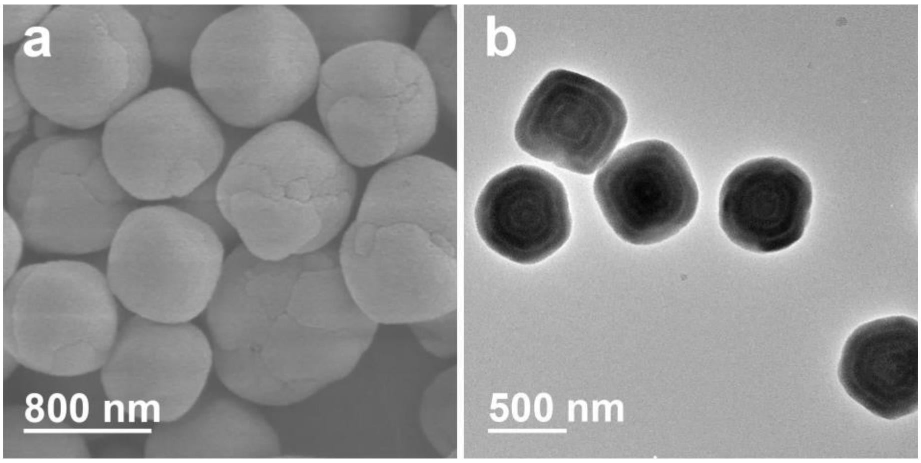

Supplementary Fig. 18 (a) SEM and (b) TEM images of UiO-66-NH_2 precursor particles with quadruple inhomogeneous layers consisting of two different mesostructured MOFs.

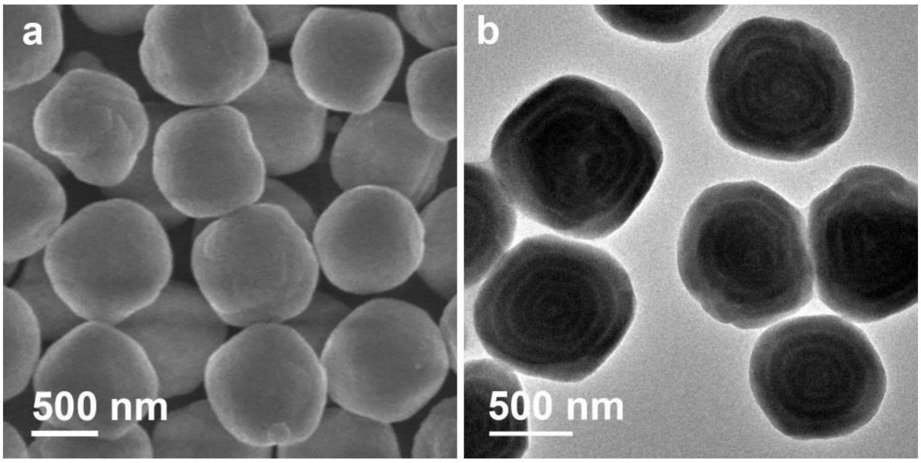

Supplementary Fig. 19 (a) SEM and (b) TEM images of UiO-66-NH_2 precursor particles with quintuple inhomogeneous layers consisting of two different mesostructured MOFs.

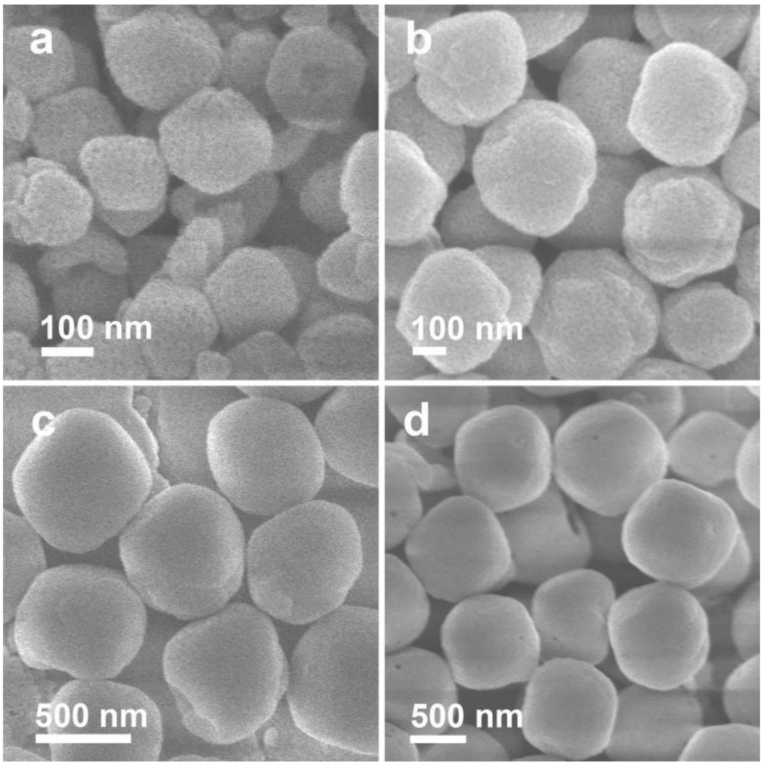

Supplementary Fig. 20 SEM images of (a) 1S-mesoUiO-66-NH_2, (b) 2S-mesoUiO-66-NH_2, (c) 4S-mesoUiO-66-NH_2, and (d) 5S-mesoUiO-66-NH_2 HoMSs.

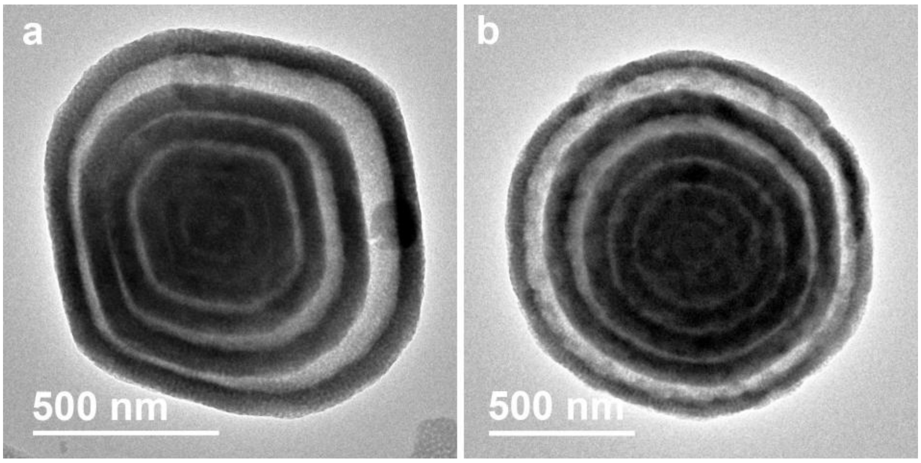

Supplementary Fig. 21 TEM images of (a) 6S-mesoUiO-66-NH_2 and (b) 7S-mesoUiO-66-NH_2 HoMSs.

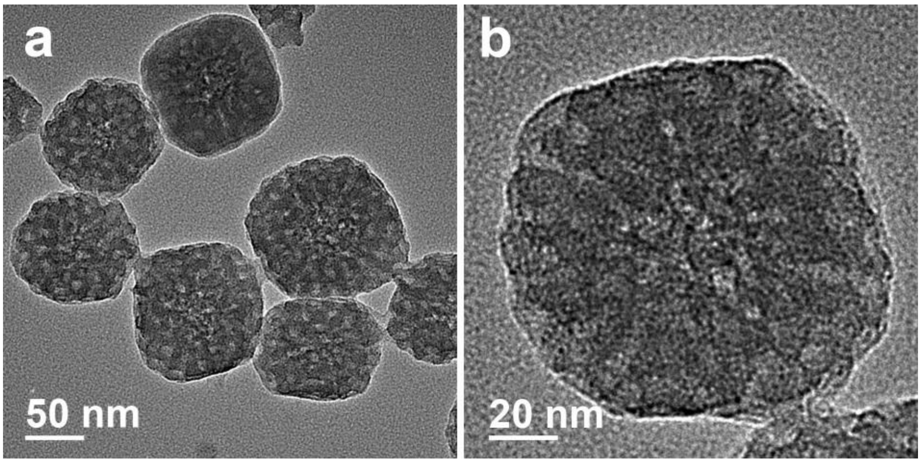

Supplementary Fig. 22 (a) TEM and (b) magnified TEM images of UiO-66-NH_2 particles prepared in 1.94 M acetic acid solution.

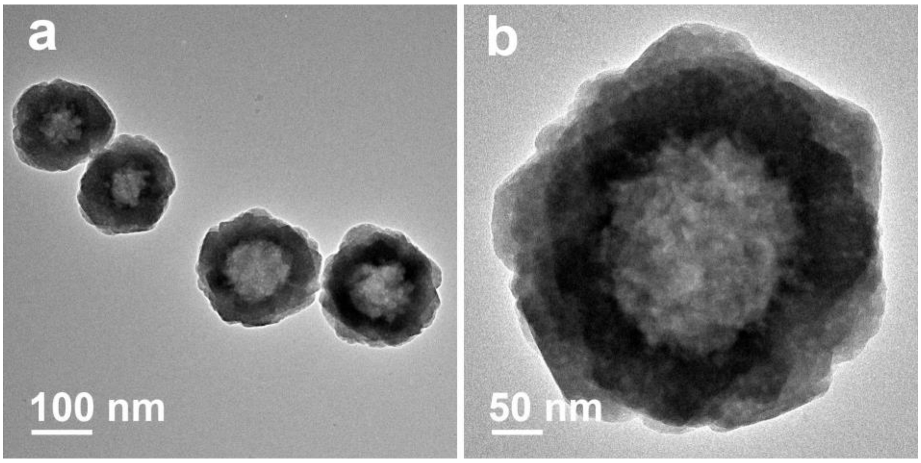

Supplementary Fig. 23 (a) TEM and (b) magnified TEM images of UiO-66-NH_2 particles prepared in 3.01 M acetic acid solution.

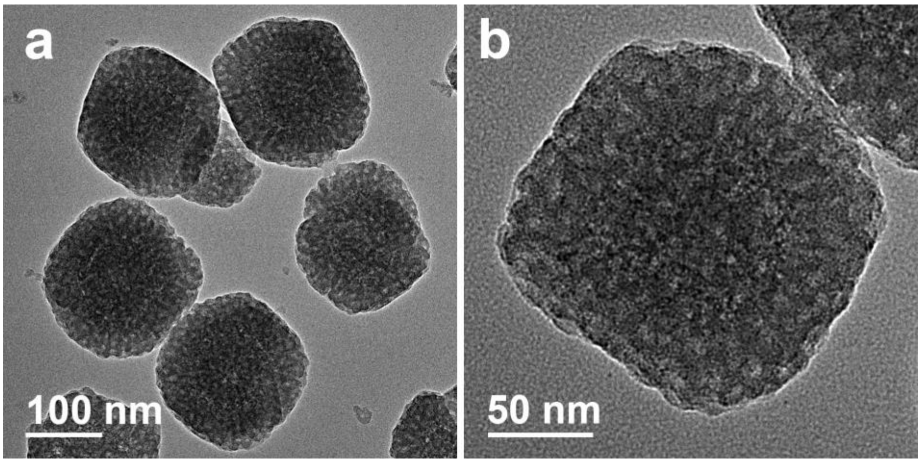

Supplementary Fig. 24 (a) TEM and (b) magnified TEM images of double-inhomogeneous-layer MOF particles synthesized by using 80% of single-layer MOF precursor particles prepared with the acetic acid concentration of 1.94 M in one pot as the cores.

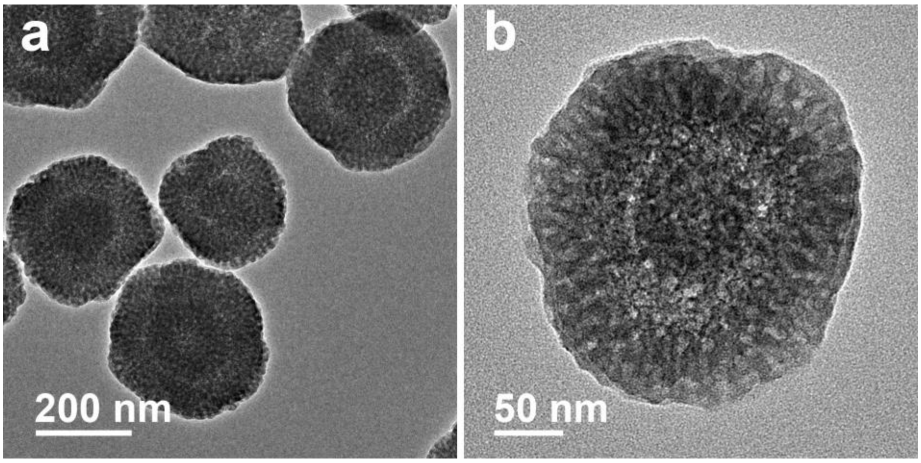

Supplementary Fig. 25 (a) TEM and (b) magnified TEM images of double-inhomogeneous-layer MOF particles prepared by using 20% of single-layer MOF precursor particles prepared with the acetic acid concentration of 1.94 M in one pot as the cores.

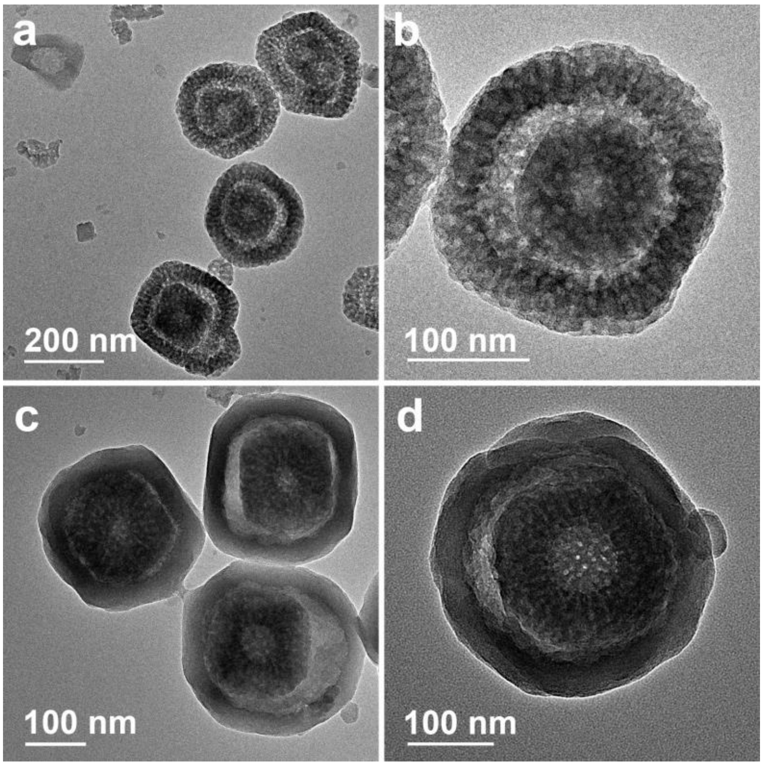

Supplementary Fig. 26 TEM images and magnified TEM images of (a,b) double-shell UiO-66-NH_2 particles with inner microporous shells and outer mesoporous shells and (c,d) double-shell UiO-66-NH_2 particles with inner mesoporous layers and outer microporous layers.

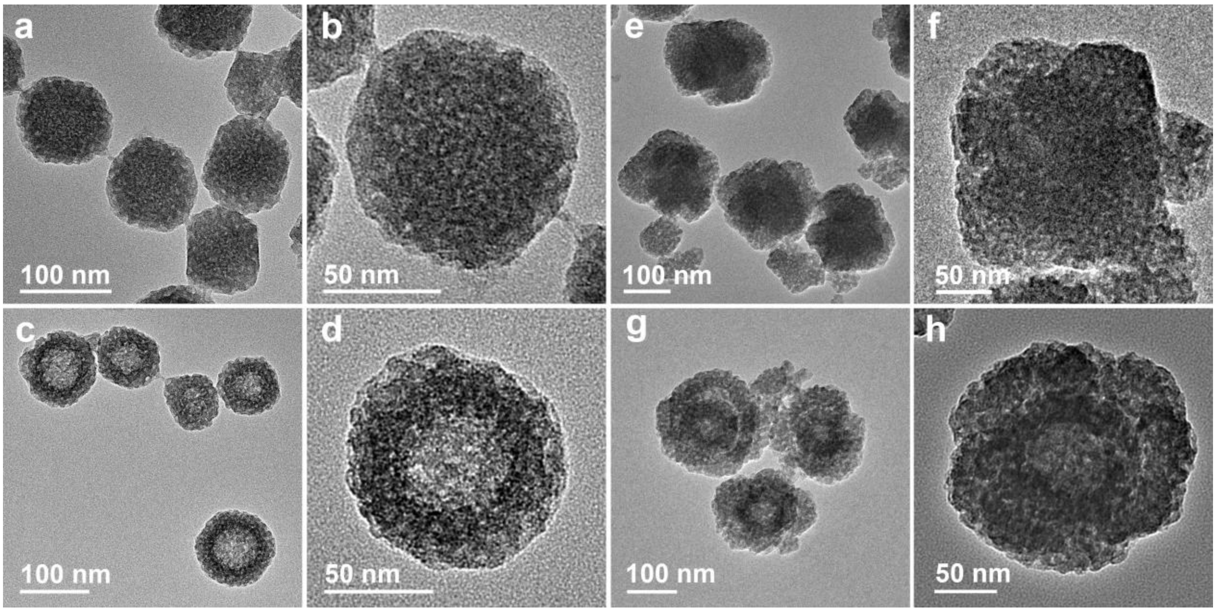

Supplementary Fig. 27 TEM images of (a,b) dual-mesopore core-shell Hf-UiO-66-NH_2 precursor particles prepared with both ODMB and F127 templates, (c,d) single-shell mesoporous Hf-UiO-66-NH_2 particles, (e,f) double-inhomogeneous-layer Hf-UiO-66-NH_2 precursor particles, and (g,h) double-shell mesoporous Hf-UiO-66-NH_2 particles.

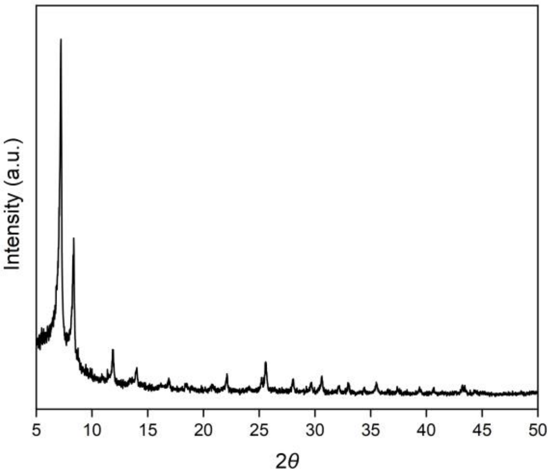

Supplementary Fig. 28 XRD pattern of double-shell mesoporous Hf-UiO-66-NH_2 particles.

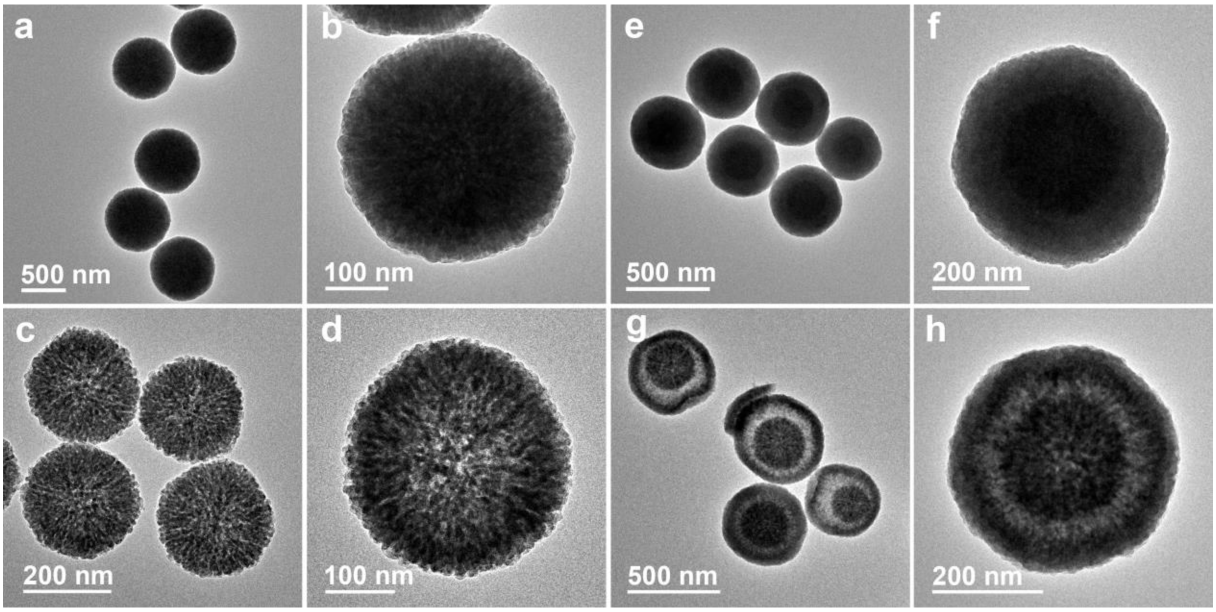

Supplementary Fig. 29 TEM images of (a,b) dual-mesopore core-shell MOF-801 precursor particles prepared with both ODMB and F127 templates, (c,d) single-shell mesoporous MOF-801 particles, (e,f) double-inhomogeneous-layer MOF-801 precursor particles, and (g,h) double-shell mesoporous MOF-801 particles.

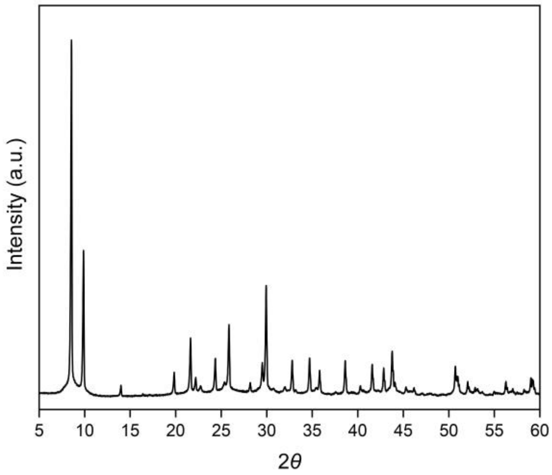

Supplementary Fig. 30 XRD pattern of double-shell mesoporous MOF-801 particles.

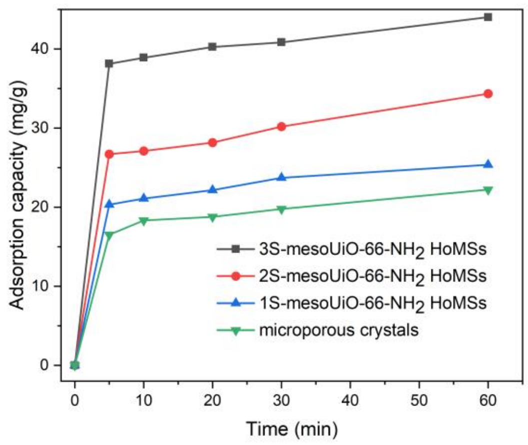

Supplementary Fig. 31 Adsorption capacity of 3S-mesoUiO-66-NH_2 HoMSs, 2S-mesoUiO-66-NH_2 HoMSs, 1S-mesoUiO-66-NH_2 HoMSs, and microporous crystals at different contact times.

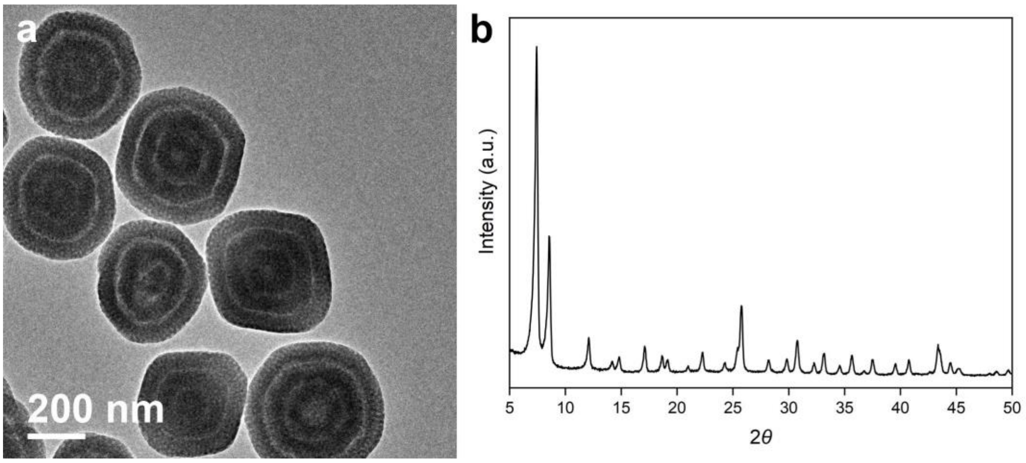

Supplementary Fig. 32 (a) TEM image and (b) XRD pattern of 3S-mesoUiO-66-NH_2 HoMSs after CO_2 cycloaddition reaction for five consecutive runs.

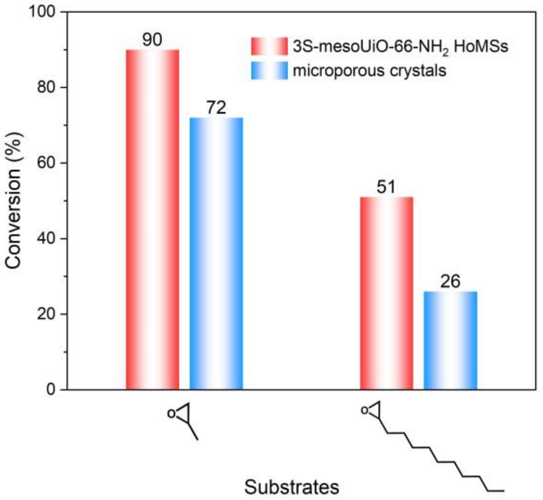

Supplementary Fig. 33 The conversion of propylene oxide and 1,2-epoxydodecane in the CO_2 cycloaddition reactions catalyzed by 3S-mesoUiO-66-NH_2 HoMSs and microporous MOF crystals.

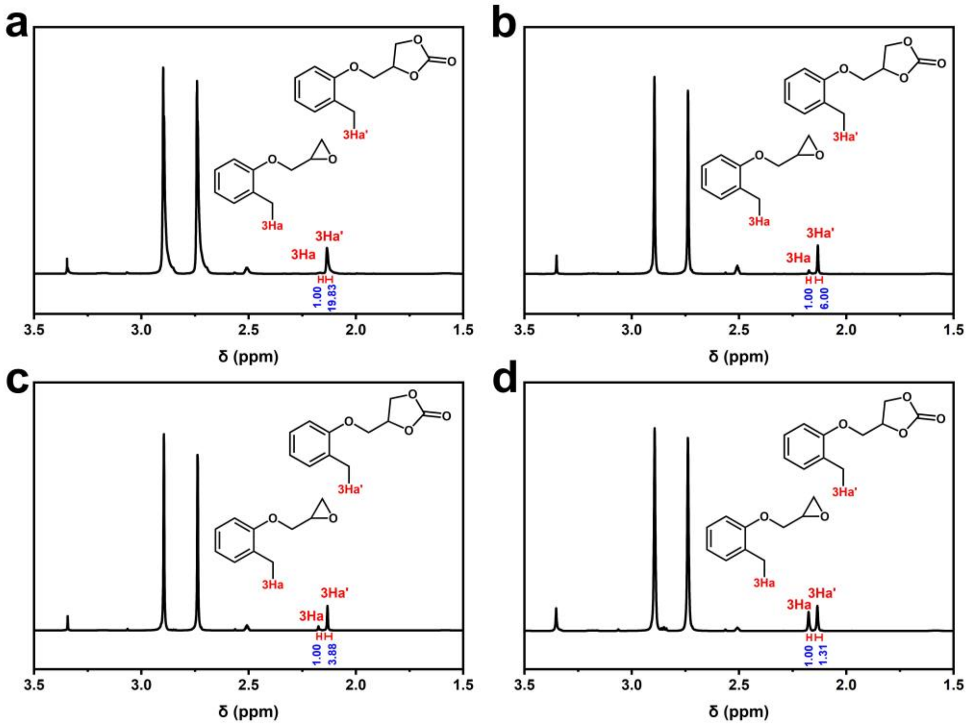

Supplementary Fig. 34 ^1H NMR spectra (DMSO-d6) of glycidyl-2-methylphenyl ether and the corresponding product under pure CO_2 atmosphere catalyzed by (a) 3S-mesoUiO-66-NH_2 HoMSs, (b) 2S-mesoUiO-66-NH_2 HoMSs, (c) 1S-mesoUiO-66-NH_2 HoMSs, and (d) microporous crystals.

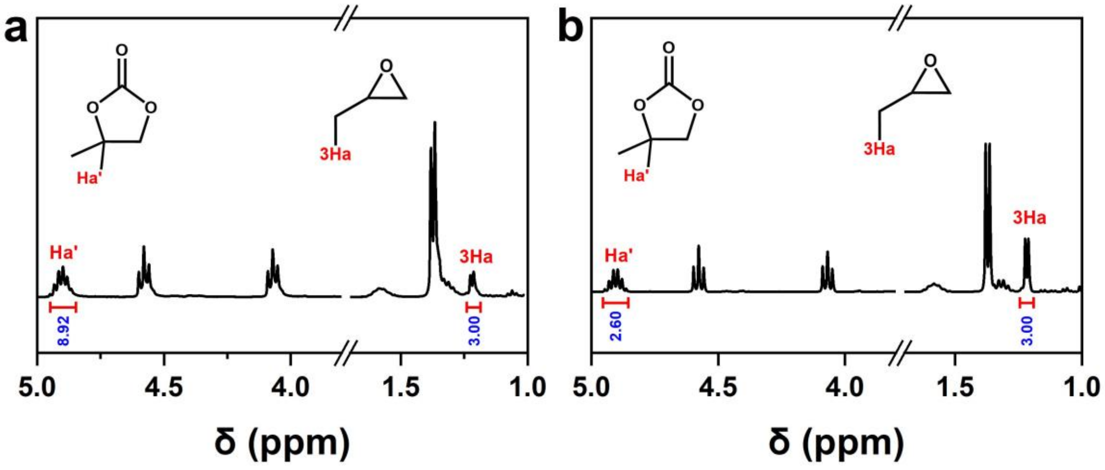

Supplementary Fig. 35 ^{1}H NMR spectra (DMSO-d6) of propylene oxide and the corresponding product under pure CO_2 atmosphere catalyzed by (a) 3S-mesoUiO-66-NH_2 HoMSs and (b) microporous crystals.

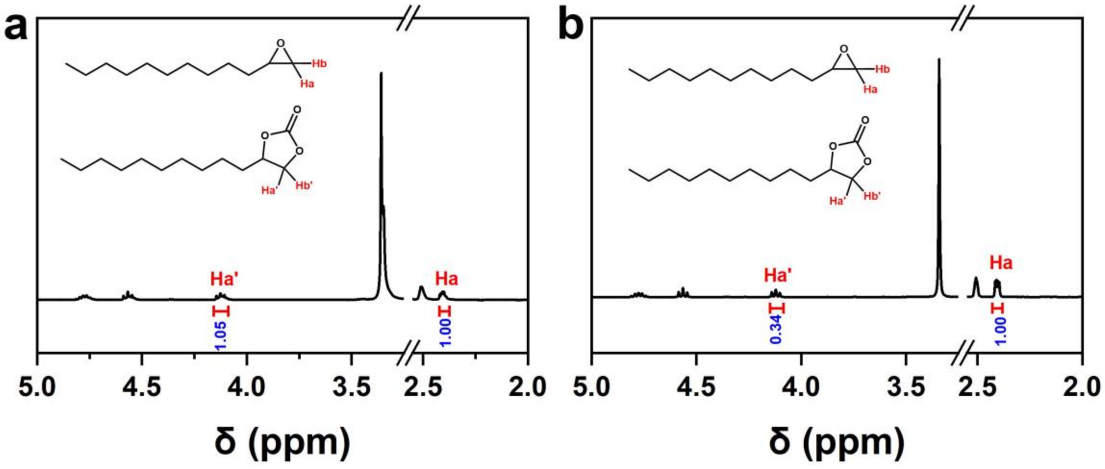

Supplementary Fig. 36 ^{1}H NMR spectra (DMSO-d6) of 1,2-epoxydodecane and the corresponding product under pure CO_2 atmosphere catalyzed by (a) 3S-mesoUiO-66-NH_2 HoMSs and (b) microporous crystals.

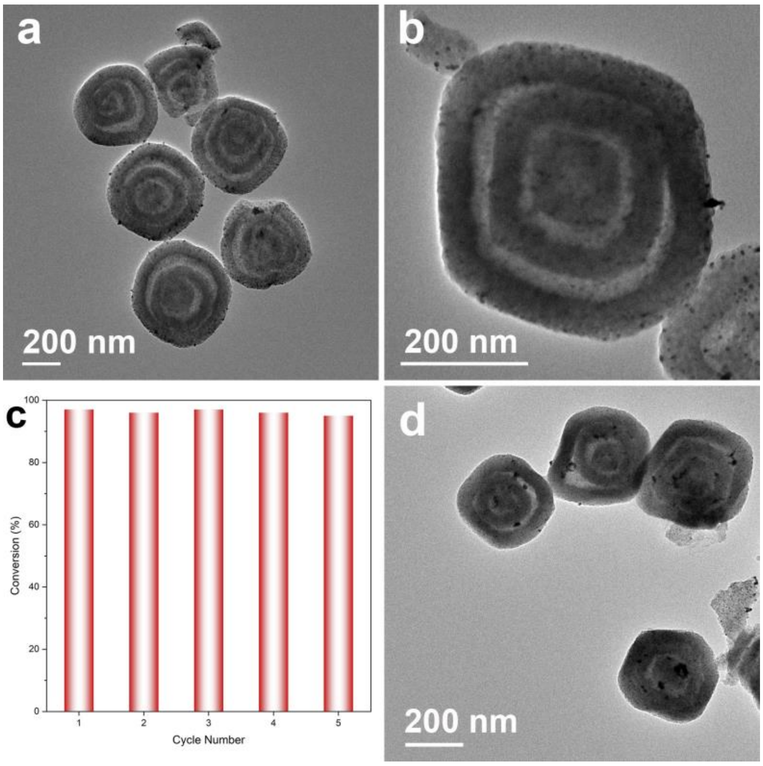

Supplementary Fig. 37 (a,b) TEM images of Au@3S-mesoUiO-66-NH_2 HoMSs, (c) the recycling tests of nitrobenzene hydrogenation over Au@3S-mesoUiO-66-NH_2 catalyst, and (d) TEM image of Au@3S-mesoUiO-66-NH_2 HoMSs after hydrogenation of nitrobenzene for five consecutive runs. **Note:** As observed from TEM images (Supplementary Fig. 37a,b), Au nanoparticles are introduced into 3S-mesoUiO-66-NH_2 HoMSs (denoting as Au@3S-mesoUiO-66-NH_2 HoMSs). The obtained nanocomposites exhibit excellent activity and recycling stability in the hydrogenation of nitrobenzene (Supplementary Fig. 37c). The particle size of Au species slight increases and the nanostructure of 3S-mesoUiO-66-NH_2 HoMSs remains unchanged after five consecutive cycles (Supplementary Fig. 37d).

Supplementary Tables

Supplementary Table 1. Comparison of 3S-mesoUiO-66-NH_2 HoMSs with other reported MOF catalysts for the CO_2 cycloaddition reactions.

Catalyst	Temperature (°C)	Pressure (bar)	Time (h)	Conversion (%)	Reference
Gea-MOF-1	120	2	6	85	*Nat. Chem.* **2014**, *6*, 673-680
FJI-H14	80	1	24	86	*Nat. Commun.* **2017**, *8*, 1233
HKUST-1	25	1	48	65	*J. Am. Chem. Soc.* **2016**, *138*, 2142-2145
(I-)Meim-UiO-66	120	1	24	33	*Chem. Sci.* **2017**, *8*, 1570-1575
PCN-222	50	4	24	66	*ChemCatChem* **2018**, *10*, 3506-3512
Co-MOF-74	100	2	4	96	*Catal. Today* **2009**, *148*, 221-231
I_{C2}HCP-5b	120	30	4	87	*ChemSusChem* **2020**, *13*, 341-350
MOF-892	25	1	60	70	*ACS Appl. Mater. Interfaces* **2018**, *10*, 733-744
In-MIL-68-NH_2	150	0.8	8	74	*ChemCatChem* **2012**, *4*, 1725-1728
ZIF-67	100	10	15	92	*New J. Chem.* **2016**, *40*, 5170-5176
3S-mesoUiO-66-NH_2	65	5	10	95	This work
